# A Unique Finding of Hepatogastric Fistula in Cervical Cancer Liver Metastasis

**DOI:** 10.7759/cureus.20761

**Published:** 2021-12-27

**Authors:** Sankar Narayanan, Pottakkat Biju, Senthamizhan Sundaramoorthy, Vishnukumar Rajaraman, Sreerekha Jinkala

**Affiliations:** 1 Surgical Gastroenterology, Jawaharlal Institute of Postgraduate Medical Education and Research, Puducherry, IND; 2 Radiation Oncology, Jawaharlal Institute of Postgraduate Medical Education and Research, Puducherry, IND; 3 Nuclear Medicine, Jawaharlal Institute of Postgraduate Medical Education and Research, Puducherry, IND; 4 Pathology, Jawaharlal Institute of Postgraduate Medical Education and Research, Puducherry, IND

**Keywords:** hepatogastric fistula, metastasis, cervical cancer, chemoradiotherapy, hepatectomy

## Abstract

In India, cervical cancer is the second leading cause of cancer-related mortality among females. Around one-third are expected to develop recurrence or metastasis during follow-up. Liver metastasis is rarely requiring palliative treatment. Patient compliance to strict follow-up is vital to detect early metastasis to be able to improve survival. A 58-year-old lady (International Federation of Gynecology and Obstetrics [FIGO] stage IIIB) was treated with concurrent chemoradiotherapy. During follow-up, she had complained of abdominal pain for which cross-sectional imaging revealed a left lobe liver lesion fistulizing into the stomach. Liver metastasis fistulizing into the stomach is a rarity, and a biopsy is required to confirm metastasis or maybe a second primary. Although palliation in the form of chemotherapy is the standard, minor or major hepatectomy can be considered in patients with good performance status at high-volume centers. A tailored multidisciplinary team approach is required for better survival.

## Introduction

Carcinoma of the cervix is the fourth most common malignancy affecting women worldwide and is a major health problem in developing countries [1]. In India, cervical cancer is the second most common malignancy in females associated with the leading cause of mortality. The number of deaths due to cervical cancer in India in 2018 was 60,078 [1-3].

Although uncommon to have metastatic disease at presentation, in 15%-61% of women with cervical cancer, metastasis to distant sites are encountered within the first two years of completing the treatment [4,5]. Hematogenous metastasis most commonly involves the lungs (36.3%) and bones (16.3%), while the liver, brain, and other sites can also be involved rarely. The average life expectancy with metastatic cervical cancer is about 12 months, and the management options are heterogeneous with palliative intent [6,7].

We report a rare presentation of the International Federation of Gynecology and Obstetrics (FIGO) stage IIIB, squamous cell carcinoma of the cervix, and post radical chemoradiation presenting with liver metastasis fistulizing into the stomach. A PubMed search did not reveal hepatogastric fistula due to cervical cancer metastases as a complication.

## Case presentation

A 58-year-old lady arrived at the emergency department with lower abdominal pain and distension. She was a known case of FIGO stage IIIB non-keratinizing squamous cell carcinoma of the cervix treated with concurrent chemoradiation. She received external beam radiation 46 Gy in 23 fractions followed by brachytherapy (8 Gy x 3 fractions prescribed to Point A) in 2019. She also received concurrent chemotherapy with weekly cisplatin 40 mg/m^2^ during radiation for five cycles.

The patient was on regular follow-up, and her disease was under remission. After eight months of completion of radiation, she presented to the emergency department with multiple episodes of upper abdominal pain. Local examination revealed a recurrent disease in the vagina, cervix, and parametrium. She also had multiple hard enlarged left supraclavicular lymph nodes with an Eastern Cooperative Oncology Group (ECOG) performance score of 2.

**Figure 1:**
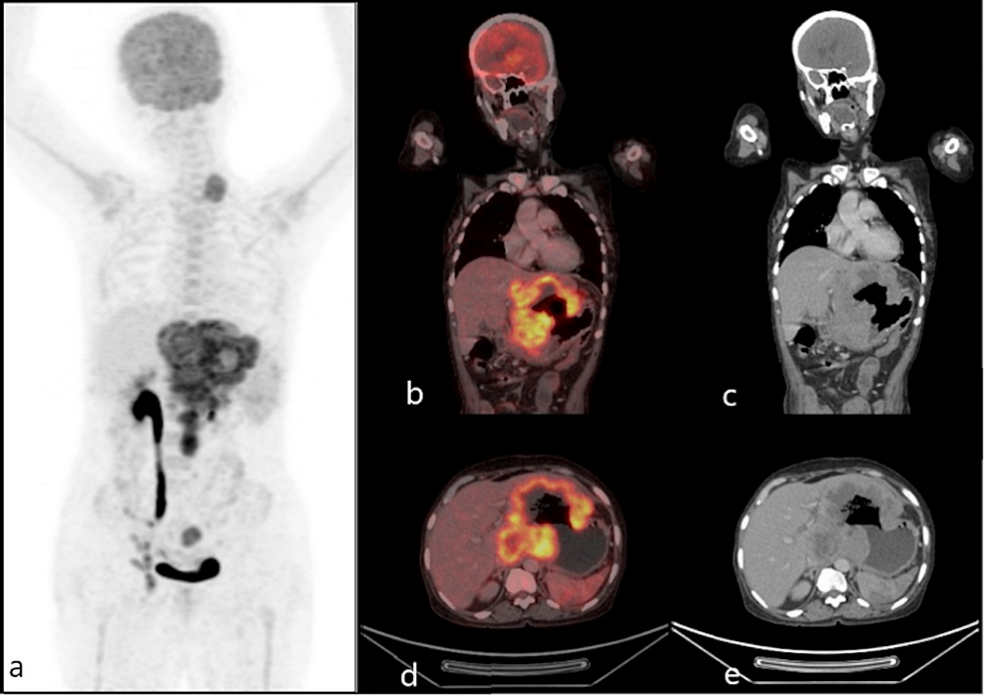
FDG PET-CT images showing (a) abnormally increased tracer uptake in the left supraclavicular region, stomach, liver, retroperitoneal region, and pelvis. Fused images (b-e) showing metabolically active lesion in segments II, III, and caudate lobes of the liver infiltrating the lesser curvature of the stomach resulting in a fistula. FDG, Fluoro-deoxy-D-glucose; PET, positron emission tomography; CT, computed tomography.

A 2-[fluorine-18] fluoro-2-deoxy-D-glucose (FDG) positron emission tomography-computed tomography (PET-CT) revealed metabolically active residual lesion posterior to the uterus extending into the pouch of Douglas measuring 2.7 cm x 2.9 cm x 5.4 cm with standardized uptake value (SUV) max of 2.22, with multiple right inguinal, pelvic, retroperitoneal and left supraclavicular lymph nodal metastases and peripyloric omental metastases (Figure 1). A metabolically active large heterogeneously enhancing lesion with necrotic areas was noted involving the segments II, III, and caudate lobes of the liver infiltrating the lesser curvature of the stomach resulting in the formation of a fistula.

**Figure 2:**
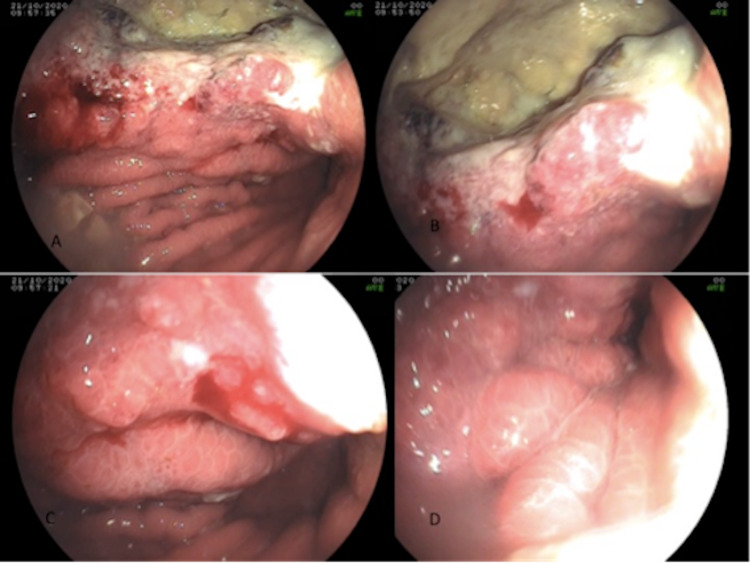
Endoscopic view showing a large necrotic lesion in the left lobe of liver forming fistula with the anterior wall of the stomach Panels (A) and (B) show the visible liver lesions from the anterior wall of the stomach with a surrounding area of necrosis. Panels (C) and (D) show the compromised stomach lumen.

As it is very unusual for a liver metastasis to produce a fistulous communication with the stomach cavity and PET-CT revealed thickened lesser curvature of the stomach, it was presumed that the patient possibly developed a second primary in the stomach with liver metastases, thus forming a fistulous communication. An upper gastrointestinal endoscopy showed a large liver lesion forming a hepatogastric fistula through the anterior wall of the stomach with areas of ulceration and necrosis. However multiple biopsies from the edge of the stomach wall close to the liver lesion confirmed metastatic squamous cell carcinoma (Figure 2).

**Figure 3:**
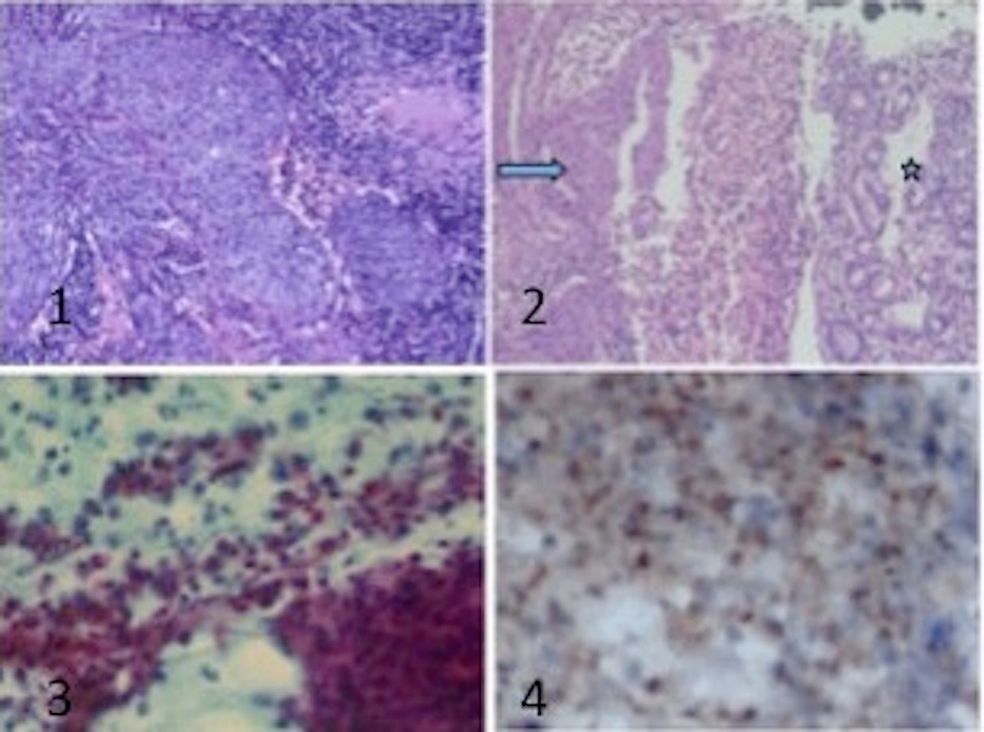
(1) H&E (200x), cervical biopsy showing tumor cells arranged in nests with abundant eosinophilic cytoplasm, vesicular to hyperchromatic nuclei with frequent mitosis consistent with SCC. (2) H&E (200x), endoscopic gastric biopsy showing normal gastric mucosa (*) along with SCC (arrow). (3) FNAC from the liver lesion showing features of metastatic SCC – Papanicolaou (200x). (4) Immunocytochemistry done on FNAC from liver lesion showing nuclear positivity in a few malignant squamous cells for p16 (DAB 400x). H&E, Hematoxylin and eosin staining; 200x, 200 times magnification; FNAC, fine needle aspiration cytology; SCC, squamous cell carcinoma; DAB, diaminobenzidine staining; 400x, 400 times magnification.

Fine needle aspiration cytology (FNAC) from the supraclavicular node also showed metastatic squamous cell carcinoma. We considered the rare possibility of a squamous cell carcinoma of the stomach in view of the asymmetric circumferential thickening of the stomach wall. In order to confirm the primary site of origin, we did a p16 immunohistochemical study on the biopsy specimen. Immunohistochemistry (IHC) was positive for p16 confirming the origin from human papillomavirus (HPV)-positive cervical carcinoma (Figure 3).

Since her biochemical parameters were within normal limits with ECOG PS of 2, she was planned for palliative chemotherapy with Inj. Paclitaxel 175 mg/m^2^ and Inj. Carboplatin AUC5 3 weekly. She completed two cycles of palliative chemotherapy. However, her condition deteriorated progressively, and she succumbed to her disease in December 2020.

## Discussion

We report a case of FIGO stage IIIB, squamous cell carcinoma of the cervix, and post radical chemoradiation presenting with liver metastasis fistulizing into the stomach. Approximately one-third of the women treated for cervical cancer will develop a recurrence during follow-up. Recurrence is classified as locoregional or distant, which usually develops within the first two years after completion of the treatment. Most of the recurrences are locoregional and located within the true pelvis or in the paraaortic nodes.

Metastases to the liver are uncommon, and the incidence of isolated liver metastasis from cervical cancer is reported to be around 1.2%-2.2% [8]. A recently published large population-based study by Zhou et al. reported the incidence of liver metastasis in cervical cancer patients around 12%, and the hematogenous metastasis most commonly involves lungs (36.3%) and bone (16.3%) [9]. Interestingly, all reported patients with liver metastasis have an uncontrolled locoregional disease. The patients with liver metastasis often present with abdominal pain, weight loss, jaundice, and vomiting. Triple-phase CT is the investigation of choice to detect liver metastases.

FDG PET-CT was validated to show an important role in staging, response assessment, evaluation of recurrence, and planning radiotherapy in carcinoma cervix. Baseline SUV max of the primary tumor, extra pelvic lymph nodes, total lesion glycolysis (TLG), and metabolic tumor volume (MTV) derived from FDG PET-CT were shown to have prognostic significance. It showed superiority over the CT and MRI for identifying recurrence at distant metastatic sites [10,11]. In our case, the PET-CT revealed a metabolically active lesion in segments 2, 3, and 1 of the liver forming a fistulous communication with the stomach cavity. The possibility of primary squamous cell carcinoma stomach and hepatocellular carcinoma (HCC) infiltrating onto the stomach was being considered in this patient. Multiple biopsies from the edge of the stomach wall, FNAC from the liver lesion, and supraclavicular lymph node confirmed metastatic squamous cell carcinoma as the pathology.

Liver metastases from cervical cancer presenting as a hepatogastric fistula are a rarity. In view of the diagnostic dilemma regarding the origin of the primary, we decided to confirm the origin using p16 immunohistochemistry, a surrogate marker for the presence of human papillomavirus infection [12]. The biopsy was positive for p16 confirming the origin of the metastasis from cervical carcinoma.

Based on the GOG (Gynecologic Oncology Group) 242 trial, the standard treatment of metastatic, persistent, or recurrent cervical carcinoma is a combination of platinum-based chemotherapy with angiogenesis inhibitor, bevacizumab [13]. The toxicity and costs of incorporating bevacizumab should be considered especially in low-resource settings. Non-anatomical wedge liver resection can be attempted in patients with limited liver disease. Adam et al. analyzed the utility of hepatic resection in the treatment of patients with non-colorectal non-endocrine liver metastases. They reported a five-year survival of 48% in patients with ovarian and uterine malignancies following hepatic resection [14]. Although the data for resection of liver metastasis from cervical cancer is sparse, minor or major hepatectomy may be attempted in patients with a limited extra pelvic disease and when the metastatic disease is well controlled or responding to systemic therapy.

## Conclusions

We report a rare case of squamous cell carcinoma of the cervix, FIGO stage IIIB, and post radical chemoradiation presenting with liver metastasis fistulizing into the stomach. The PET-CT is recommended in the initial stage and during the follow-up of such patients. The role of hepatectomy is safe in the selected individuals at high-volume centers.
